# Bioremediation potential of *Pseudomonas aeruginosa *and *Enterobacter cloacae *isolated from a copper-contaminated area

**DOI:** 10.1186/1753-6561-8-S4-P188

**Published:** 2014-10-01

**Authors:** Marcela Baltazar, Louise Gracioso, Ingrid Avanzi, Marcela Veiga, Luciana Gimenes, Claudio Nascimento, Elen Perpetuo

**Affiliations:** 1 University of São Paulo, Chemical Engineer Department, PQI-POLI-USP Center for Environmental Research and Training, CEPEMA-POLI-USP, SP, Brazil; 2 University of São Paulo, Biomedical Sciences Institute, ICB-USP Center for Environmental Research and Training, CEPEMA-POLI-USP, SP, Brazil; 3 Center for Environmental Research and Training, CEPEMA-POLI-USP, SP, Brazil; 4 University of São Paulo, Biomedical Sciences Institute, ICB-USP University of São Paulo, Chemical Engineer Department, PQI-POLI-USP Center for Environmental Research and Training, CEPEMA-POLI-USP, SP, Brazil

## Background

The Sossego mine, located in Canaã dos Carajás, Pará, Brazil, has a pond of wastes with low copper concentrations economically unfeasable for extraction. In this place, we can improve environmental conditions and, at the same time, recover part of the ore diluted in these wastes, through evaluation and use of the local biodiversity, in bioremediation processes, once the use of this technology, will allow the decontamination as well the recovery of these metals with high value.

Once there are some restrictions on the microorganisms introduction in the environment, it is important to establish the bioremediation potential of native species from a particular location. Therefore, it is necessary to study the biodegradation processes or biotransformation of compounds in the microbial biodiversity already adapted that are responsible for these processes in the environment, first in a bench scale[1].

In this work, among the 22 strains isolated from environmental samples from a copper mine, two of them presented great potential for bioremediation. Strains were identified and both were subjected to comparative study of their bioremediation potential and showed good results in concentrations up to 320ppm of copper.

## Methods

Strains were identified by mass spectrometry (MALDI-TOF-Biotyper). Both strains were grown in 150ml DYP medium supplemented with 8g/L of casamino acids and in parallel with the same medium, added of 160 and 320ppm of copper in flasks of 500mL incubated in an orbital shaker at 28 ° C and 200 rpm. Samples were taken along 24h (one in one hour) and monitored for cellular growth and copper biosorption by spectrophotometer UV-Vis (600 nm) and atomic absorption spectroscopy (AA), respectively. As a control of copper adsorbed, samples were taken at 0h and 24h and analyzed also by AA. At the end of exponential phase of cellular growth, the biomass was utilized to construct a correlation curve between absorbance and dry mass of the cells.

## Results and conclusion

Metals removal using micro-organisms selected has become very promising, since they can exhibit high selectivity and rate of removal, and also has advantage of having the potential to regenerate biomass, allowing reuse in the further steps, after the metal recovery[2-4].

In this work, most efficient strains for bioremediation of effluents contaminated by copper were identified by mass spectrometry as *Pseudomonas aeruginosa *and *Enterobacter cloacae *presenting a high score for species identification (2,43 and 2,24 respectively). *P.aeruginosa *showed high tolerance to concentrations up to 160ppm of copper and *E.cloacae *up to 320ppm of copper. Considering the current conditions of the Sossego mine, *P.aeruginosa *and *E.cloacae *could be very efficient in the reusing process of copper available in the pond. Moreover, the strains isolated in this study showed better results than those described in the literature^5^. *P.aeruginosa *was able to remove 30% of copper from a medium containing 160ppm of copper compared with 23% of removal described by Sethuraman & Kumar[5], and *E.cloacae *adsorbed 50% of 320 ppm of copper, compared to 20% in the same study[5].
